# Safety of clinical engineer-assisted percutaneous coronary intervention

**DOI:** 10.1007/s12928-022-00884-w

**Published:** 2022-08-09

**Authors:** Mitsutoshi Oguri, Hideki Ishii, Takuro Shigematsu, Rin Fujita, Yuichiro Koyama, Takeshi Katagiri, Yoshihiro Ikai, Yusuke Fujikawa, Hiroshi Takahashi, Yoriyasu Suzuki, Toyoaki Murohara

**Affiliations:** 1 Department of Cardiology, Kasugai Municipal Hospital, 1-1-1 Takaki-cho, Kasugai, Aichi 486-8510 Japan; 2 Department of Cardiovascular Medicine, Gunma University Graduate School of Medicine, Maebashi, Japan; 3 Division of Medical Statistics, Fujita Health University, Toyoake, Japan; 4 Department of Cardiology, Nagoya Heart Center, Nagoya, Japan; 5 Department of Cardiology, Nagoya University Graduate School of Medicine, Nagoya, Japan

**Keywords:** Clinical engineer, Percutaneous coronary intervention, Safety

## Abstract

Percutaneous coronary intervention (PCI) requires multiple staff members, including interventional cardiologists, with the physical burden of heavy protective measures to minimize radiation exposure. Here, we aimed to investigate the safety of task sharing with clinical engineers (CEs) working as 1st assistant during ad hoc PCI. We retrospectively included 286 patients who underwent ad hoc PCI following diagnostic catheterization for coronary artery disease between April 2019 and March 2021. Procedural complications including coronary perforation or rupture, myocardial infarction, cerebral embolism, cardiovascular death, decreased kidney function, and radiation parameters were compared between the two clinical settings [CE group, CEs as the 1st assistant from the beginning of diagnostic coronary angiography to the end of PCI vs. doctor (DR) group, others]. There was no increase in the ratio of procedural complications in the CE group (1.7%) versus the DR group (1.2%). Fluorescence time and radiation exposure dose were significantly reduced in the CE group {25 min [interquartile range (IQR), 19–35 min] vs. 28 min (IQR, 20–39 min), *P* = 0.036; 908 mGy (IQR, 654–1326 mGy) vs. 1062 mGy (IQR, 732–1594 mGy), *P* = 0.049}. The median amount of contrast medium was significantly reduced in the CE group [100 mL (IQR, 80–119 mL) vs. 110 mL (IQR 90–140 mL), *P* < 0.001]. After propensity matching, fluorescence time, radiation exposure dose, and contrast medium amount were similar between groups. Task sharing with CEs as the 1st assistant during ad hoc PCI could contribute to clinical safety in patients with coronary artery disease.

## Introduction

Percutaneous coronary intervention (PCI), already established as a standard and beneficial strategy for the treatment of patients with coronary artery disease [1, 2], requires multiple staff members including interventional cardiologists, nurses, clinical engineers (CEs), and radiological technologists in the catheterization room. PCI performed electively or after diagnostic catheterization usually takes 1–2 h. Two or more interventional cardiologists routinely engage as operators and assistants during PCI and carry the physical burden while taking heavy protective measures to minimize radiation exposure.

Robotic remote-controlled assisted PCI has recently safely and effectively replaced the physical burden of interventional cardiologists [3]. However, it may not be possible to widely introduce such an ideal and expensive system. Apart from promoting advanced technology, the Ministry of Health, Labour and Welfare in Japan proposed the introduction of a task-sharing system, shifting some tasks to other occupations, as a realizable strategy in real clinical settings (https://www.mhlw.go.jp/content/10800000/000720006.pdf). With this background, the proposal of task sharing with CEs playing a supportive role during cardiac catheterizations, including diagnostic coronary angiography and PCI, was officially approved in our hospital to reduce the workload of interventional cardiologists. Thereafter, we developed and implemented a training program for CEs separated by procedure to maintain clinical safety. Thus, the present study aimed to compare any differences in clinical safety between the two clinical settings (CEs as the 1st assistant and others) in patients with coronary artery disease.

## Methods

### Study population

**Fig. 1:**
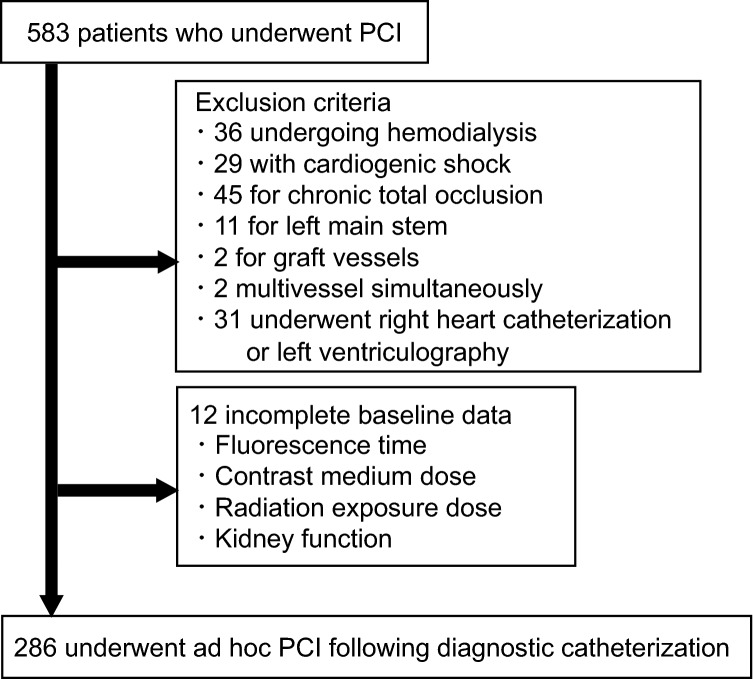
The study flowchart. *PCI* percutaneous coronary intervention

Among 583 patients who underwent PCI, the retrospective study finally enrolled 286 patients who underwent ad hoc PCI following diagnostic catheterization for coronary artery disease at Kasugai Municipal Hospital (Kasugai, Japan) from April 2019 to March 2021 (Fig. 1). In cases of planned PCI, devices and treatment strategies were decided in advance by our heart team. Therefore, since there may be little difference in clinical safety with or without CE assistance, we excluded those patients who underwent planned PCI. Noninvasive anatomical or functional assessments are frequently conducted before coronary angiography; therefore, ad hoc PCI can be adapted to some cases at the physician’s discretion according to the guidelines [4]. Patients undergoing hemodialysis; those with cardiogenic shock with or without mechanical devices; and those who underwent PCI for chronic total occlusion, a left main stem coronary artery, graft vessels, or multivessel simultaneously were excluded. Patients who underwent PCI after right heart catheterization or left ventriculography were also excluded because fluorescence time, radiation exposure dose, or contrast medium dose could differ between patients. The primary outcome of the study was procedural complications including coronary perforation or rupture, myocardial infarction, cerebral embolism, cardiovascular death, decreased kidney function. The secondary outcomes included fluorescence time, radiation exposure dose, contrast medium amount, and procedural time. The study protocol complied with the Declaration of Helsinki and was approved by the Committee on Ethics of Kasugai Municipal Hospital. We also offered the opportunity to opt out to all patients. (https://www.hospital.kasugai.aichi.jp/byouin/torikumi/rinsho/rinri/documents/rinri_461.pdf); however, none of the subjects decided to opt out.

### Catheterization procedure

All procedures were performed using standard coronary catheterization and interventional techniques. The radial approach is usually recommended; however, the femoral or brachial approach is sometimes performed in limited cases of insufficient flow of the ulnar artery or difficulty advancing a wire or catheter. For diagnostic coronary angiography, 4 Fr sheaths and catheters are generally used with 2000 units of unfractionated heparin through a peripheral vein. Before the ad hoc PCI, additional unfractionated heparin was administered. In patients with a diagnosis of ST-elevation myocardial infarction, 6 or 7 Fr sheaths were inserted from the beginning with an appropriate dose of unfractionated heparin. Contrast medium was administered manually, and a biplane angiogram was used for every patient. Catheters and camera angles used during fluoroscopy and cineangiography were selected at the operator’s discretion. Fluoroscopic and cineangiographic images were acquired at 15 flames/s. Fluorescence time and radiation exposure dose were calculated from the beginning of diagnostic coronary angiography to the end of PCI using the Allura Clarity FD 10/10 RoHS coronary angiography system (PHILIPS, Amsterdam, the Netherlands). The amount of contrast medium used for the procedure and procedural time were also measured from the beginning of diagnostic coronary angiography to the end of PCI.

Seven CEs, without practical experience of coronary angiography or PCI prior to this study, participated in the present study. CEs help actual PCI operation processes along with practice guidelines published by the Japan Association for Clinical Engineers (http://ja-ces.or.jp/wordpress/01jacet/shiryou/pdf/2012gyoumubetsu_gyoumushishin06.pdf). The practice guidelines recommend coverage for the approved tasks and limitations, in which the injection of contrast medium and radiation application are not allowed for CEs. The training program was prepared based on both practice guidelines and procedural characteristics listed in the Japanese Percutaneous Coronary Intervention Registry [5] (Table 1). All CEs underwent three trainings based on this program per item.

**Table 1:** Training program

	Process	Detailed contents
1	In-deflator preparation	Prepare an in-deflator with 5 mL of contrast media and 5 mL of saline
2	Sheath introducer preparation	Confirm with raising a voice, and flush with saline
3	Sheath exchange	Assist operator during exchanging the sheath, if needed
4		Confirm with raising a voice for additional heparin introduction by nurse
5	Guide catheter preparation	Flush with saline and connect with the Y-connecting tube
6	Guide catheter advance	Assist operator during advance of the guide catheter
7	Reference images storage	Store reference images according to operator’s instruction
8	Prepare coronary guidewire(s)	Be sure to raise a voice before taking it out from the case. And then, flush with saline
9	Intravascular ultrasound or optimal coherence technology preparation	Be sure to raise a voice before taking out from the case, then flush with saline and check if it works
10	Thrombus aspiration catheter preparation	Be sure to raise a voice before removing it from the case. Flush with saline
11	Balloon preparation	Be sure to repeat the exact size with raising a voice before taking out from the case. Connect with an in-deflator and remove air
12	Balloon operation (including drug-coated balloon and scoring balloon)	Inflate following operator’s instruction. Be sure to repeat the indicated pressure exactly with raising a voice. Check the fluorescent image if the balloon dilates while checking the pressure of in-deflator
13	Stent preparation	Be sure to repeat the exact size with raising a voice before taking out from the case Connect with an in-deflator and remove air (before or after advancing to the lesion)
14	Stent operation	Inflate following operator’s instruction. Be sure to repeat the indicated pressure exactly with raising a voice. Check the fluorescent image if the stent dilates while checking the pressure of in-deflator
15	Review	Review the PCI process with other stuff in the catheterization room

*PCI* percutaneous coronary intervention

### Definition

Acute myocardial infarction (MI) is defined based on fourth universal definition of MI [6]. Procedural complications include coronary perforation or rupture, myocardial infarction, cerebral embolism, and cardiovascular death. Decreased kidney function was defined as an impairment in kidney function resulting in an increase from baseline in serum creatinine ≥ 0.3 mg/dL on the day after PCI.

### Data collection

Patients’ clinical characteristics (age, sex, smoking status, body mass index, previous medical history, non-cardiac comorbidities, procedural information, and angiographic and radiological parameters) and PCI-related complications were assessed by chart review.

### Statistical analysis

Categorical variables are expressed as counts and percentages, while continuous variables are expressed as median and interquartile range (IQR) or mean ± standard deviation. Categorical variables were compared using the chi-square test. Continuous variables were compared using the unpaired Student’s *t* test (for normally distributed variables) or the Mann–Whitney *U* test (for skewed variables). Characteristics and clinical outcomes were compared between the CE group (CEs as the 1st assistant from the beginning of the diagnostic coronary angiography to the end of PCI) and the doctor (DR) group (doctors as the 1st assistant or CEs and doctors assisting together during the procedure). To minimize selection bias for CE assistance, a propensity-matched analysis was performed. First, to obtain the propensity score, a multivariate logistic regression analysis was performed using the model including CEs as the 1st as assistant as the dependent variable. Age; sex; body mass index; presence of diabetes, hypertension, dyslipidemia, previous myocardial infarction, multivessel disease, or a bifurcation lesion; experienced operator; diseased vessel (right, left anterior descending, or left circumflex coronary artery); and access site (radial artery or others) were included as independent variables. Second, the propensity scores for individual cases were calculated using the logistic model. Statistical significance was set at *P* < 0.05. SAS software (version 27; SAS Institute, Inc. Cary, NC, USA) was used for the statistical analyses.

## Results

The characteristics of the enrolled patients categorized based on the 1st assistant of their index PCI, are shown in Table 2. The prevalence of dyslipidemia and previous MI was greater, whereas the ratio of acute MI and experienced operators was smaller in the CE group than in the DR group. Other variables, including angiographic characteristics, were similar between the two groups. In the DR group, 11 doctors attended as assistants, with their clinical experiences of ≥ 3– < 5 years (*N* = 4), ≥ 5– < 10 years (*N* = 3), and ≥ 10 years (*N* = 4), and 39 cases were performed with one CE and one doctor as assistants. There was no increase in the ratio of procedural complications in the CE group (1.7%) versus the DR group (1.2%) (Table 3). The fluorescence time and radiation exposure dose were significantly reduced in the CE group [25 min (IQR, 19–35 min) vs. 28 min (IQR, 20–39 min), *P* = 0.036; 908 mGy (IQR, 654–1326 mGy) vs. 1062 mGy (IQR, 732–1594 mGy), *P* = 0.049]. The median procedural time was 90 min (IQR, 75 − 120 min) for the CE group and 105 min (IQR, 80 − 126) min (*P* = 0.155) for the DR group. Additionally, the median amount of contrast medium used for the procedure was significantly reduced in the CE group [100 mL (IQR, 80–119 mL) vs. 110 mL (IQR, 90–140 mL), *P* < 0.001] in the DR group. Serum creatinine levels before and on the day after PCI and the ratio of decrease in kidney function were similar.

**Table 2:** Characteristics of the study subjects

	CE group *n* = *116*	DR group *n* = 170	*P* value
Age, years	72 (64 − 79)	70 (56 − 79)	0.181
Male sex, %	69.0	77.7	0.100
Current or former smoker, %	51.7	56.5	0.429
Body mass index, kg/m2	24.3 (22.1 − 26.6)	24.2 (22.1 − 26.3)	0.471
Dyslipidemia, %	77.6	61.2	0.004
Type 2 diabetes mellitus, %	44.8	42.9	0.752
Hypertension, %	86.2	78.8	0.112
Chronic heart failure, %	6.0	10.0	0.282
Previous myocardial infarction, %	25.0	13.5	0.014
Coronary bypass surgery, %	0.9	0.6	1.000
Diagnosis (AMI/others), %	37.9/62.1	60.6/39.4	< 0.001
Experience of operator in PCI (< 5 years/ ≥ 5 to < 10 years/ ≥ 10 years), %	3.5/56.0/40.5	15.3/49.4/35.3	0.006
Multiple vessel disease, %	41.4	50.6	0.125
Coronary lesions			0.264
Left anterior descending artery, %	48.3	56.5	0.173
Right coronary artery, %	30.2	21.8	0.108
Left circumflex artery, %	20.7	21.8	0.828
ACC/AHA lesion type B2 or C, %	44.0	40.6	0.570
Bifurcation lesion, %	16.4	16.5	1.000
Radial artery access, %	94.0	90.6	0.379
Imaging device use, %	98.3	100	0.164

Categorical variables are described as percentages, and continuous variables are presented as median and 25–75th percentile range

*ACC/AHA* American College of Cardiology/American Heart Association, *AMI* acute myocardial infarction, *CE* clinical engineer, *DR* doctor, *PCI* percutaneous coronary intervention

**Table 3:** Clinical outcomes

	CE group *n* = *116*	DR group *n* = 170	*P* value
Procedural complications, %	1.7	1.2	1.000
Coronary perforation or rupture, n	1	1	
Myocardial infarction, n	1	0	
Cerebral embolism, n	0	1	
Cardiovascular death, n	0	0	
Fluorescence time, min	25 (19–35)	28 (20–39)	0.036
Radiation exposure dose, mGy	908 (654–1326)	1062 (732–1594)	0.049
Procedural time, min	90 (75–120)	105 (80–126)	0.155
Contrast medium dose, mL	100 (80–119)	110 (90–140)	< 0.001
*Serum creatinine, mg/dL*			
Before PCI	0.88 (0.72–1.06)	0.88 (0.76–1.02)	0.832
The day after PCI	0.83 (0.68–0.97)	0.82 (0.73–0.98)	0.521
Decreased kidney function, *n* (%)	2 (1.7)	2 (1.2)	1.000

Categorical variables are described as percentages, while continuous variables are presented as median and 25–75th percentiles

*CE* clinical engineer, *DR* doctor, *PCI* percutaneous coronary intervention

Sixty-six patients from each group were matched according to their estimated propensity scores. The patients’ baseline clinical and angiographic characteristics are listed in Table 4. Procedural complications were rare, with only one case in the DR group. Fluorescence time, radiation exposure dose, contrast medium amount, and procedural time were similar between groups (Table 5). The ratio of a decrease in kidney function was also similar between groups.

**Table 4:** Characteristics of the study subjects after propensity score matching

	CE group *n* = *66*	DR group *n* = 66	*P* value
Age, years	72 (61 − 79)	74 (58 − 81)	0.578
Male sex, %	75.8	78.8	0.836
Current or former smoker, %	51.5	59.1	0.381
Body mass index, kg/m2	24.6 (21.6 − 26.1)	23.5 (21.1 − 24.8)	0.130
Dyslipidemia, %	72.7	74.2	1.000
Type 2 diabetes mellitus, %	34.9	39.4	0.589
Hypertension, %	81.8	86.4	0.635
Chronic heart failure, %	6.1	7.6	1.000
Previous myocardial infarction, %	12.1	16.7	0.621
Diagnosis (AMI/others), %	48.5/51.5	47.0/53.0	0.862
Experience of operator in PCI (< 5 years/ ≥ 5 to < 10 years/ ≥ 10 years), %	6.1/54.5/39.4	4.6/54.5/40.9	0.922
Multiple vessel disease, %	48.5	54.6	0.486
Coronary lesions			0.915
Left anterior descending artery, %	53.0	51.5	0.862
Right coronary artery, %	25.8	24.2	1.000
Left circumflex artery, %	21.2	24.2	0.836
ACC/AHA lesion type B2 or C, %	45.5	33.3	0.154
Bifurcation lesion, %	13.6	15.2	1.000
Radial artery access, %	93.9	93.9	1.000
Imaging device use, %	98.5	100	1.000

Categorical variables are described as percentages, and continuous variables are presented as median and 25th–75th percentiles

*ACC/AHA* American College of Cardiology/American Heart Association *AMI* acute myocardial infarction, *CE* clinical engineer, *DR* doctor, *PCI* percutaneous coronary intervention

**Table 5:** Clinical outcomes in propensity score matched groups

	CE group *n* = *66*	DR group *n* = 66	*P* value
Procedural complications, %	0	1.5	1.000
Coronary perforation or rupture, *n*	0	0	
Periprocedural myocardial infarction, *n*	0	0	
Cerebral embolism, *n*	0	1	
Cardiovascular death, *n*	0	0	
Fluorescence time, min	26 (18 − 35)	27 (19 − 38)	0.794
Radiation exposure dose, mGy	915 (672 − 1408)	960 (668 − 1444)	0.928
Procedural time, min	100 (79 − 120)	102 (75 − 120)	0.848
Contrast medium dose, mL	100 (80 − 125)	110 (85 − 130)	0.703
*Serum creatinine, mg/dL*			
Before PCI	0.90 (0.72 − 1.08)	0.91 (0.77 − 1.09)	0.530
The day after PCI	0.88 (0.66 − 1.03)	0.84 (0.73 − 1.00)	0.739
Decreased kidney function, *n* (%)	2 (3.0)	2 (3.0)	1.000

Categorical variables are described as percentages, and continuous variables are presented as median and 25th–75th percentiles

*CE* clinical engineer, *DR* doctor, *PCI* percutaneous coronary intervention

Detailed instructions for each CE were required at the beginning of the study, which may have influenced the clinical outcomes. Therefore, we compared each variable between the two groups by dividing them into two periods (April 2019–March 2020 and April 2020–March 2021) (Table 6). Regarding the clinical outcomes, there was no intergroup difference in the early and late periods.

**Table 6:** Patients’ clinical outcomes by period in propensity score matched groups

	CE group *n* = *46*	DR group *n* = 42	*P* value	CE group *n* = 20	DR group *n* = *24*	*P* value
Period	From April 2019 to March 2020			From April 2020 to March 2021		
Procedural complications, %	0	2.4	1.000	0	0	N/A
Contrast medium dose, mL	100 (80–126)	110 (89–120)	0.997	98 (83–124)	105 (81–140)	0.508
Fluorescence time, min	25 (18–33)	25 (17–39)	0.920	32 (17–36)	28 (23–35)	0.934
Radiation exposure dose, mGy	905 (661–1343)	867 (550–1416)	0.605	959 (695–1564)	981 (818–1744)	0.588
Procedural time, min	100 (75–123)	103 (73–120)	0.654	98 (80–120)	98 (80–120)	0.868

Procedural complications included coronary perforation and rupture, myocardial infarction, cerebral embolism, and cardiovascular death

*CE* clinical engineer, *DR* doctor, *N/A* not applicable

## Discussion

This single-center retrospective study suggested that the task-sharing system with CEs by working as the 1st assistant during ad hoc PCI ensures clinical safety in terms of the equal occurrence of procedural complications and equivalent radiological parameters. To the best of our knowledge, the present study is the first to investigate clinical outcomes and thereby evaluate the safety of CE assistance during ad hoc PCI. Although relatively small in number, this task-sharing system could reduce the physical burden of interventional cardiologists while maintaining safety.

Coronavirus disease 2019 (COVID-19) has emerged as a pandemic worldwide; therefore, interventional cardiology including acute coronary syndrome treatment was strongly affected [7, 8]. Many reports showed that elective PCI and other procedures required suspension or postponing, and significant reduction in the incidence of MI requiring intervention was observed [9, 10]. Even if we follow standard precaution with protective equipment, infection in the catheterization laboratory is possible. For PCI in definite or highly probable COVID-19 patients, the number of staff in the catheterization laboratory should be restricted to prevent outbreaks [11]. In this regard, our commitment to promoting task sharing with CEs could be suitable for COVID-19 hospitals. Additionally, it may be noteworthy to replenish doctors who were forced to manage COVID-19, including treating patients or providing vaccinations in the community.

In a Japanese nationwide registry, the risk of in-hospital adverse events increased with age, and the rate of in-hospital mortality among septuagenarians was 1.56% in patients with acute coronary syndrome and 0.09% in those with stable coronary artery disease [12]. Similar trends were observed in other registries with a large number of patients undergoing PCI [13]. We experienced a case of coronary perforation by wiring (but not cardiac tamponade) and a case of periprocedural MI due to a distal embolism after ballooning in the CE group. However, no case was presumed directly affected by CE assistance. Al-Mukhaini et al. reported that potentially lethal complications including coronary perforation or rupture occur at an incidence of 0.19–0.59% among various complications during PCI [14]. In addition, procedural complications, especially coronary perforation or rupture, may occur if inappropriate assistance for advancing wiring or devices or non-compliance to the instructions in ballooning occurs. However, such serious events did not occur in our study, probably corresponding to their training progress. Furthermore, although we experienced a small number of procedural complications, an equivalent difference was observed between the two groups.

Radiation exposure is indispensable for coronary angiography and PCI procedures; however, it may be harmful to the patient. Therefore, we should reduce radiation exposure doses in clinical practice along with the revision of the guidelines on Radiation Safety in Cardiology, indicating growing interest in minimizing radiation exposure for the prevention of health damage [15]. We should monitor the radiation dose for each patient based on distance, time, and shield, and medical staff should wear protective clothing. With regard to time, the fluoroscopy-save function, standard equipped to the radiation system, may play a pivotal role. In particular, CEs were repeatedly recommended to use the fluoroscopy-saving function as much as possible in our training program. To our experiences, doctors sometimes tend to step on the foot pedal for saving an imaging. Therefore, CEs were expected to interrupt such an additional radiation exposure using the fluoroscopy-saving function during PCI. In our study, fluorescence time and radiation exposure were effectively reduced in the CE group among the selected patients in the crude analysis. Given that there were significant differences in fluorescence time (*P* = 0.004) and radiation exposure (*P* < 0.001) according to operator experience, infrequent attendance of CEs to PCI with young operators may have affected the results. Furthermore, fluorescence time per procedural time was still significantly longer in PCI with younger operators (*P* = 0.022). In this respect, it is difficult to estimate the impact of fluoroscopy-saving function usage; CEs will have a change to contribute to PCI with young operators using the fluoroscopy-saving function.

Radial artery access is recommended for patients with acute MI as well as chronic coronary syndrome [4]. Prior studies have provided clinical evidence of the reduction of complications and lower radiation exposure [16, 17]. In our study, most patients underwent the radial artery approach (94.0% in the CE group vs. 90.6% in the DR group). In accordance with clinical safety, the unified method may have affected our results.

The present study had some limitations: (i) the results presented here are only from a single hospital with a relatively small sample size. Although we used propensity-matched scores, there was a high chance of bias in many of the data assignments or that with other variables not included in propensity-matched scores. In addition, it is impossible to provide guaranty in case of serious complications due to the small number of complications. Thus, future analyses with a larger sample size and randomization are necessary. (ii) We excluded patients with serious or infrequent conditions. However, there was also a bias in case selection. (iii) It was impossible to adjust the clinical experience of each operator or CE. (iv) In the present study, we could not separate the radiation parameters for diagnostic angiography from the total time or dose. (v) It was difficult to estimate the precise effect of task-sharing system on the efficacy of overall catheterization laboratory and physician workload because of the lack of survey or detail of overtime work hours of each physician.

In conclusion, this study’s findings suggest that the task-sharing system, the CE as the 1^st^ assistant during ad hoc PCI, could contribute to clinical safety in patients with coronary artery disease. Therefore, our study results are clinically important and may serve as the groundwork in this field.
